# 3D convolutional neural networks-based multiclass classification of Alzheimer’s and Parkinson’s diseases using PET and SPECT neuroimaging modalities

**DOI:** 10.1186/s40708-021-00144-2

**Published:** 2021-11-02

**Authors:** Ahsan Bin Tufail, Yong-Kui Ma, Qiu-Na Zhang, Adil Khan, Lei Zhao, Qiang Yang, Muhammad Adeel, Rahim Khan, Inam Ullah

**Affiliations:** 1 School of Electronics and Information Engineering, Harbin Institute of Technology, Harbin, 150001 China; 2 Department of Electrical and Computer Engineering, COMSATS University Islamabad, Sahiwal Campus, Sahiwal, Pakistan; 3 Department of Computer Science, University of Peshawar, Peshawar, Pakistan; 4 Didi Chuxing, Beijing, China; 5 Institute of Space Technology, Islamabad, Pakistan; 6 Hohai University, Nanjing, China

**Keywords:** Dementia, Neuroimaging modalities, Pattern recognition, Deep learning, Discrete cosine transform

## Abstract

**Background:**

Alzheimer’s disease (AD) is a neurodegenerative brain pathology formed due to piling up of amyloid proteins, development of plaques and disappearance of neurons. Another common subtype of dementia like AD, Parkinson’s disease (PD) is determined by the disappearance of dopaminergic neurons in the region known as substantia nigra pars compacta located in the midbrain. Both AD and PD target aged population worldwide forming a major chunk of healthcare costs. Hence, there is a need for methods that help in the early diagnosis of these diseases. PD subjects especially those who have confirmed postmortem plaque are a strong candidate for a second AD diagnosis. Modalities such as positron emission tomography (PET) and single photon emission computed tomography (SPECT) can be combined with deep learning methods to diagnose these two diseases for the benefit of clinicians.

**Result:**

In this work, we deployed a 3D Convolutional Neural Network (CNN) to extract features for multiclass classification of both AD and PD in the frequency and spatial domains using PET and SPECT neuroimaging modalities to differentiate between AD, PD and Normal Control (NC) classes. Discrete Cosine Transform has been deployed as a frequency domain learning method along with random weak Gaussian blurring and random zooming in/out augmentation methods in both frequency and spatial domains. To select the hyperparameters of the 3D-CNN model, we deployed both 5- and 10-fold cross-validation (CV) approaches. The best performing model was found to be AD/NC(SPECT)/PD classification with random weak Gaussian blurred augmentation in the spatial domain using fivefold CV approach while the worst performing model happens to be AD/NC(PET)/PD classification without augmentation in the frequency domain using tenfold CV approach. We also found that spatial domain methods tend to perform better than their frequency domain counterparts.

**Conclusion:**

The proposed model provides a good performance in discriminating AD and PD subjects due to minimal correlation between these two dementia types on the clinicopathological continuum between AD and PD subjects from a neuroimaging perspective.

## Introduction

Alzheimer’s disease (AD) is a widely spread subtype of dementia and a major target for healthcare applications. It is an irremediable and progressive disease with millions of cases worldwide. Staggering costs are associated with the management of AD due to which this disease remains a focal point of healthcare authorities worldwide. The brain parts that are normally affected during the course of progression of AD are hippocampus, lateral ventricle, insula, putamen, entorhinal cortex, lingual gyrus, amygdala, thalamus, supramarginal gyrus, caudate nucleus, uncus, etc. [1, 2].

Parkinson’s disease (PD) is another brain disorder that is affecting millions of people worldwide and has variable prevalence rates with aged individuals getting affected substantially in comparison to younger counterparts just like AD. This disease is defined by neuronal loss in the region known as substantia nigra pars compacta located in the midbrain and formation of neuromelanin. PD is prevalent in both males and females with higher prevalence in males. It affects speech resulting in dysarthria, hypophonia, tachyphemia, etc., affecting the voice of an individual [3, 4].

Both AD and PD are incurable diseases, but medication is available to keep the symptoms under control [5]. Coexistence of both these subtypes of dementia is possible in the presence of visual hallucinations, sleep behavior disorder, fluctuations in attention or cognition, tau phosphorylation, inflammation, and synaptic degeneration [6].

Deep learning methods are widely deployed in the literature for classification, action recognition, speech recognition as well as other tasks, etc. These methods are extremely good at learning features that optimally represent data for the problem at hand. They tend to act like black boxes where information is processed by keeping the operator of the loop. Features learned by Convolutional Neural Networks (CNNs) are known to possess invariance, equivariance and equivalence properties. Architectures such as 3D-CNNs can extract both spectral and spatial domain features simultaneously from the input volume. The building blocks of these architectures are convolutional layer, pooling layer, batch normalization, dropout regularization as well as fully connected layer, etc. [7].

In the literature, studies have been proposed for the classification tasks such as AD vs Normal Control (NC), progressive mild cognitive impairment (pMCI) vs static mild cognitive impairment (sMCI), pMCI vs NC using a combination of modalities such as magnetic resonance imaging (MRI), positron emission tomography (PET), functional MRI as well as other modalities and non-imaging data such as ApoE genotype, cerebrospinal fluid (CSF) concentration of Aβ_1–42_, Mini-Mental State Examination (MMSE), Alzheimer’s Disease Assessment Scale-Cognitive subscale (ADAS-Cog), Rey Auditory Verbal Learning Test (RAVLT), Functional Assessment Questionnaire (FAQ) Neuropsychiatric Inventory Questionnaire (NPI-Q), etc., using different deep learning models [8–10].

Similarly for PD diagnosis, research has been conducted using voice datasets [11, 12], using isosurfaces-based features, using statistics-based learning methods, to discover hidden patterns of PD using CNNs, and also using neuromelanin-sensitive MRI modality achieving high performance on assessment metrics [13]. While learning features in the spatial domain using CNNs has its own advantages, learning in the frequency domain might offer advantages that spatial domain methods are unable to provide. In the CNN models, low-frequency domain components are better learned than the higher ones offering advantages such as better preservation of image information in the pre-processing stage as well as other advantages [14]. Discrete Cosine Transform (DCT) is a frequency domain method often used to define a sequence of data points using cosine functions offering advantages in terms of compactness of information.

Data augmentation methods such as adversarial techniques improve the performance of models expanding limited datasets so as to enable them to expand their generalization power.

There is a growing body of works available in the literature to study correlation between different types of dementia. David Irwin et al. [15] examined PD cases along with correlates of co-morbid AD confirming that there is an abundance of AD pathology in PD subjects which may result in modifying the clinical phenotype. As a matter of fact, co-morbid AD is also strongly associated with the changes in PD suggesting a potential clinicopathological continuum between AD and PD.

To add to the growing body of works available in the literature for understanding the clinicopathological continuum between co-morbid AD and PD cases from a neuroimaging perspective, this research effort is aimed at studying correlation between these two dementia subtypes using PET and SPECT neuroimaging modalities and deep learning methods for joint multiclass classification task.

In this work, we utilized both spatial and frequency (DCT) domain methods to learn features extracted from whole-brain images of PET scans and single photon emission computed tomography (SPECT) scans of AD and PD subjects using a 3D-CNN architecture. We deployed random weak Gaussian blurring and random zoomed in–out as data augmentation methods individually and in combination. Different from other studies in the literature where focus is on the binary or multiclass classification of AD or PD subjects, we focused on the joint multiclass classification of both AD and PD subjects and extracted features from whole-brain image scans using 3D-CNN architectures.

Rest of the paper is organized as follows. A description of the datasets is given in Sect. 2, methodology in Sect. 3, experiments in Sect. 4, results and their discussion in Sect. 5, and finally, conclusion in Sect. 6.

## Datasets description

We used PPMI [16] and ADNI databases [17] for the experiments. We utilized 3D-SPECT scans from the PPMI database and 3D-PET scans from the ADNI database. Demographics of the subjects considered for this study are given in Table 1 and Table 2.

**Table 1:** Demographics of subjects with PET scans presented in mean (min–max) format

Research group	NC	AD
Number of subjects	102	94
Age	76.01 (62.2–86.6)	75.82 (55.3–88)
Weight	75.7 (49–130.3)	74.12 (42.6–127.5)
FAQ total score	0.186 (0–6)	13.67 (0–27)
NPI-Q total score	0.402 (0–5)	4.074 (0–15)

**Table 2:** Demographics of subjects with SPECT scans presented in mean (min–max) format

Research group	NC	PD
Number of subjects	94	99
Gender	Females: 31, males: 63	Females: 40, males: 59
Age	65.97 (50–84)	66.49 (50–85)

## Methodology

**Fig. 1:**
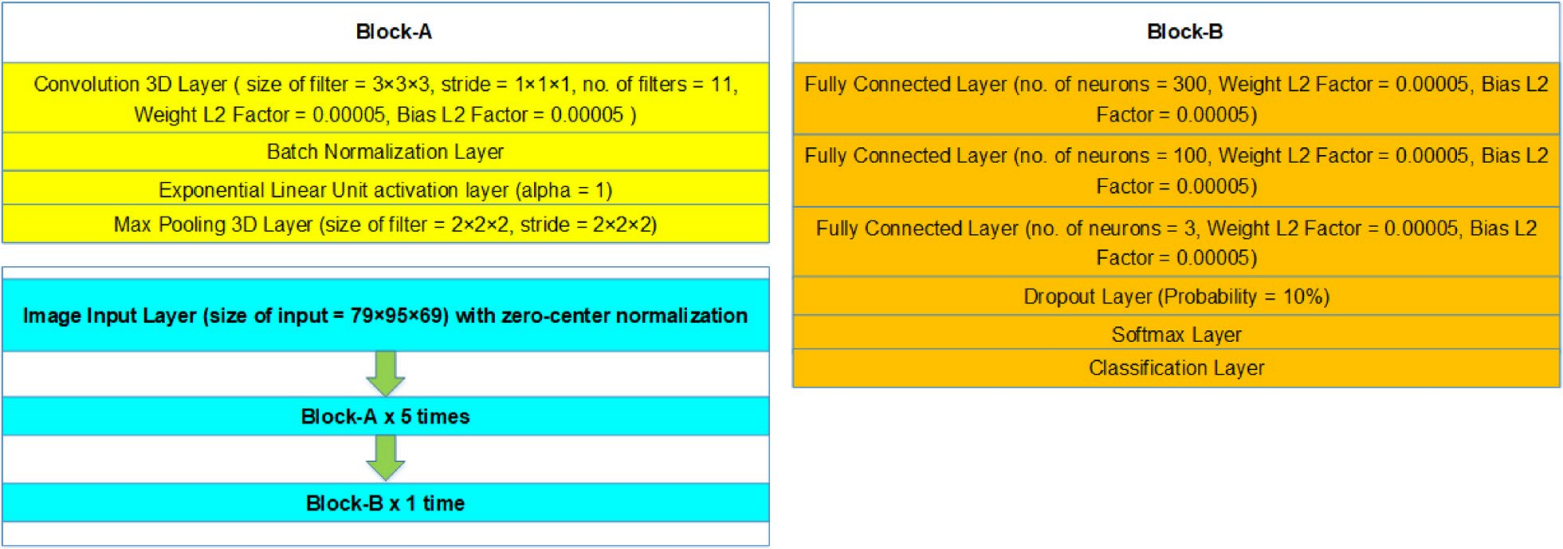
Schematic diagram of the 3D-CNN architecture

To carry out the experiments for the joint multiclass (3-classes) classification between AD, PD and NC classes, we deployed a 3D-CNN architecture for all the experiments as shown in Fig. 1. An input layer accepts a volume of size 79 × 95 × 69 normalized through zero-center procedure. It works by dividing each channel with its standard deviation, subtracting the mean in the process to center the volume towards the origin. Then, a convolutional layer extracts the features from this volume. The tensor of convolutional filter weights is dependent on the number of channels, temporal depth, width and height of the filter. Mathematically, this process can be defined as:

1 $$v_{ij}^{abc} = f\left( {b_{ij} + \mathop \sum \limits_{m} \mathop \sum \limits_{p = 0}^{{P_{i} - 1}} \mathop \sum \limits_{q = 0}^{{Q_{i} - 1}} \mathop \sum \limits_{r = 0}^{{R_{i} - 1}} w_{ijm}^{pqr} w_{{m\left( {i - 1} \right)}}^{{\left( {a + p} \right)\left( {b + q} \right)\left( {c + r} \right)}} } \right),$$

where *P*_*i*_, *Q*_*i*_, *R*_*i*_ are the kernel sizes along the three dimensions, respectively. $v_{ij}^{abc}$ is the value of the (*a*, *b*, *c*)th element of the *j*th feature map in the *i*th layer, $w_{ijm}^{pqr}$ denotes the value of (*p*, *q*, *r*)th element of the 3D convolution kernel connected to the *m*th feature map.

Stride of the layer is another important parameter that represents the number of pixels skipped during the convolution operation. Setting its value to one convolves every input pixel with the kernel. Another important parameter is L2 regularization also known as weight decay that is used to drive the weights towards the origin without making them exactly zero. We add L2 regularization to convolutional and fully connected layers to help the network in avoiding over-fitting. Batch normalization technique is then used on the mini-batches to effectively avoid over-fitting and for speeding up the learning process. We then used Exponential Linear Unit (ELU) as activation function for nonlinear mapping of inputs. Mathematically, it can be defined as:

2 $${\text{ELU}}:\;\left\{ {\begin{array}{*{20}c} {x, x \ge 0} \\ {\alpha \left( {e^{x} - 1} \right), x < 0} \\ \end{array} } \right..$$

We deployed max pooling to learn effective fast representations of inputs by reducing the dimensions. After that, we deployed dense or fully connected layers and a dropout layer with a probability of 10%. Finally, a combination of softmax and classification layers classify the input into one of the three classes: AD, PD and NC. Table 3 shows our detailed architecture hyperparameters.

**Table 3:** Architecture hyperparameters for our proposed 3D-CNN model

Layer	Filter size	Number of filters	Stride size	Dropout rate	Output size
Conv1 + BN + ELU	3 × 3 × 3	11	1	–	11 × 79 × 95 × 69
MaxPool1	2 × 2 × 2	–	2	–	11 × 40 × 48 × 35
Conv2 + BN + ELU	3 × 3 × 3	11	1	–	11 × 40 × 48 × 35
MaxPool2	2 × 2 × 2	–	2	–	11 × 20 × 24 × 18
Conv3 + BN + ELU	3 × 3 × 3	11	1	–	11 × 20 × 24 × 18
MaxPool3	2 × 2 × 2	–	2	–	11 × 10 × 12 × 9
Conv4 + BN + ELU	3 × 3 × 3	11	1	–	11 × 10 × 12 × 9
MaxPool4	2 × 2 × 2	–	2	–	11 × 5 × 6 × 5
Conv5 + BN + ELU	3 × 3 × 3	11	1	–	11 × 5 × 6 × 5
MaxPool5	2 × 2 × 2	–	2	–	11 × 3 × 3 × 3
FC 1	–	300	–	–	300
FC 2	–	100	–	–	100
FC 3	–	3	–	–	3
Dropout	–	–	–	0.1	3
Softmax	–	–	–	–	3

*BN* batch normalization; *Conv* convolutional layer; *FC* fully connected; *MaxPool*max pooling

In the model design, to mitigate the internal covariance shift between the batch normalization and dropout techniques, we added only one dropout layer right before the softmax layer to reduce over-fitting since there are no following batch normalization layers. We add five max-pooling layers to our model with kernel and stride size 2 × 2 × 2 in order to scale down the output feature map size by a factor of 2^5^ compared with the original input.

We used small spatial receptive field of size 3 × 3 × 3 to increase the performance of our convolutional layers and we add padding in these layers. The softmax layer is a 1 × 1 convolutional layer followed by softmax activation. Our model is designed efficiently to get maximum performance while offering less number of computations.

## Experiments

We performed splitting at the subject level for the experiments for the multiclass classification task. We deployed two data augmentation methods: (1) random weak Gaussian blurring and (2) random zooming in/out. We set the *σ* value to 1.5 for the random weak Gaussian blurring, and scale value to 0.99 and 1.03 for the random zooming in/out augmentations to decrease as well as increase the size of the input volumes at random. We performed experiments in the spatial and frequency (DCT) domain without augmentation and in the presence of single and combined augmentation methods. Here combined augmentations are methods that used samples of both random weak Gaussian blurred augmentation and random zoomed in/out augmentation in the training set. We used augmented samples for training purposes and non-augmented samples for validation and test purposes.

We deployed fivefold and tenfold CV procedures for the experiments on balanced datasets. We also created a test subset, and in this subset, we placed 4 samples of NC(SPECT) class, 9 samples of PD class, 4 samples of AD class, and 12 samples of NC(PET) class.

Other settings are as follows. Mini-batch is set to a size of 2, initial learning rate is set to 0.001, maximum number of epochs are set to 30, and optimizer is set to Adam. We run a total of 170 experiments and completed all of our simulations in approximately 3242 min or 54.03 h.

## Results and discussion

The methods and results of the experiments are presented in Tables 4, 5, 6, 7 and 8, respectively. We used Relative Classifier Information (RCI), Confusion Entropy (CEN), Index of Balanced Accuracy (IBA), Geometric Mean (GM), and Matthew’s Correlation Coefficient (MCC) as our performance assessment metrics. A description of methods employed in the study is given in Table 4. The Serial # column in Table 4 through Table 8 is the same.

**Table 4:** A description of methods employed in the study

Serial #	Methods
1	AD/NC(SPECT)/PD classification with random weak Gaussian blurred augmentation in the spatial domain using fivefold CV approach
2	AD/NC(SPECT)/PD classification with random weak Gaussian blurred augmentation in the spatial domain using tenfold CV approach
3	AD/NC(SPECT)/PD classification with combined augmentations in the spatial domain using tenfold CV approach
4	AD/NC(SPECT)/PD classification with random zoomed in/out augmentation in the frequency domain using fivefold CV approach
5	AD/NC(SPECT)/PD classification with combined augmentations in the frequency domain using tenfold CV approach
6	AD/NC(SPECT)/PD classification with random zoomed in/out augmentation in the spatial domain using fivefold CV approach
7	AD/NC(SPECT)/PD classification with random weak Gaussian blurred augmentation in the frequency domain using fivefold CV approach
8	AD/NC(SPECT)/PD classification with combined augmentations in the spatial domain using fivefold CV approach
9	AD/NC(SPECT)/PD classification with random zoomed in/out augmentation in the spatial domain using tenfold CV approach
10	AD/NC(SPECT)/PD classification with combined augmentations in the frequency domain using fivefold CV approach
11	AD/NC(SPECT)/PD classification without augmentation in the frequency domain using fivefold CV approach
12	AD/NC(SPECT)/PD classification without augmentation in the frequency domain using tenfold CV approach
13	AD/NC(SPECT)/PD classification without augmentation in the spatial domain using fivefold CV approach
14	AD/NC(SPECT)/PD classification with random weak Gaussian blurred augmentation in the frequency domain using tenfold CV approach
15	AD/NC(SPECT)/PD classification with random zoomed in/out augmentation in the frequency domain using tenfold CV approach
16	AD/NC(SPECT)/PD classification without augmentation in the spatial domain using tenfold CV approach
17	AD/NC(PET)/PD classification without augmentation in the spatial domain using fivefold CV approach
18	AD/NC(PET)/PD classification without augmentation in the spatial domain using tenfold CV approach
19	AD/NC(PET)/PD classification without augmentation in the frequency domain using fivefold CV approach
20	AD/NC(PET)/PD classification without augmentation in the frequency domain using tenfold CV approach

**Table 5:** RCI, CEN and IBA performance metrics for the methods

Serial #	RCI	CEN			IBA		
		AD	NC	PD	AD	NC	PD
1	0.916	0	0.093	0.091	1	0.958	0.921
2	0.9046	0	0.1071	0.1022	1	0.9171	0.9404
3	0.9046	0	0.1071	0.1022	1	0.9171	0.9405
4	0.8976	0	0.1124	0.1115	1	0.9576	0.8829
5	0.8862	0	0.1255	0.1236	1	0.9372	0.8831
6	0.885	0	0.129	0.123	1	0.897	0.921
7	0.8848	0	0.1281	0.1243	1	0.9171	0.9021
8	0.885	0	0.128	0.124	1	0.917	0.902
9	0.8848	0	0.1282	0.1243	1	0.9171	0.9021
10	0.8675	0	0.1458	0.1436	1	0.9169	0.8646
11	0.862	0	0.149	0.15	1	0.937	0.828
12	0.858	0	0.1568	0.1533	1	0.897	0.8647
13	0.858	0	0.156	0.153	1	0.897	0.864
14	0.8491	0	0.167	0.1622	1	0.8773	0.8647
15	0.8412	0	0.1749	0.1712	1	0.8773	0.8463
16	0.8332	0	0.1863	0.1759	1	0.8195	0.883
17	0.7519	0.279	0.265	0	0.729	0.748	1
18	0.7464	0.2859	0.2702	0	0.7116	0.7483	1
19	0.7308	0.3054	0.2838	0	0.6605	0.7478	1
20	0.6266	0.3272	0.3924	0.0983	0.6279	0.61	0.998

**Table 6:** GM and MCC performance metrics for the methods

Serial #	GM			MCC		
	AD	NC	PD	AD	NC	PD
1	1	0.98	0.974	1	0.953	0.954
2	1	0.9708	0.9742	1	0.9445	0.9462
3	1	0.9708	0.9742	1	0.9445	0.9462
4	1	0.9738	0.964	1	0.9378	0.9381
5	1	0.9685	0.9615	1	0.9296	0.9304
6	1	0.963	0.966	1	0.93	0.93
7	1	0.9657	0.964	1	0.929	0.9304
8	1	0.965	0.964	1	0.929	0.93
9	1	0.9657	0.964	1	0.929	0.9304
10	1	0.9606	0.9537	1	0.914	0.915
11	1	0.96	0.946	1	0.908	0.907
12	1	0.9552	0.9511	1	0.9057	0.9071
13	1	0.955	0.951	1	0.905	0.907
14	1	0.9498	0.9485	1	0.8975	0.8995
15	1	0.9473	0.9433	1	0.8899	0.8916
16	1	0.936	0.9457	1	0.8806	0.8854
17	0.8898	0.8945	1	0.7814	0.79	1
18	0.8842	0.892	1	0.773	0.7832	1
19	0.8673	0.8845	1	0.7477	0.7628	1
20	0.8528	0.8333	0.9778	0.7198	0.678	0.9361

**Table 7:** Average values of CEN, IBA, GM and MCC performance metrics for the methods

Serial #	Average CEN	Average IBA	Average GM	Average MCC
1	0.0613	0.9597	0.9847	0.969
2	0.0698	0.9525	0.9817	0.9636
3	0.0698	0.9525	0.9817	0.9636
4	0.0746	0.9468	0.9793	0.9586
5	0.083	0.9401	0.9767	0.9533
6	0.084	0.9393	0.9763	0.9533
7	0.0841	0.9397	0.9766	0.9531
8	0.084	0.9397	0.9763	0.953
9	0.0842	0.9397	0.9766	0.9531
10	0.0965	0.9272	0.9714	0.943
11	0.0997	0.9217	0.9687	0.9383
12	0.1034	0.9206	0.9688	0.9376
13	0.103	0.9203	0.9687	0.9373
14	0.1097	0.914	0.9661	0.9323
15	0.1154	0.9079	0.9635	0.9272
16	0.1207	0.9008	0.9606	0.922
17	0.1813	0.8257	0.9281	0.8571
18	0.1854	0.82	0.9254	0.8521
19	0.1964	0.8028	0.9173	0.8368
20	0.2726	0.7453	0.888	0.778

**Table 8:** Ranking of the methods

Serial #	RCI-based ranking	CEN-based ranking	IBA-based ranking	GM-based ranking	MCC-based ranking	Overall score	Overall ranking
1	1	1	1	1	1	5	1
2	2	2	2	2	2	10	2
3	2	2	2	2	2	10	2
4	3	3	3	3	3	15	3
5	4	4	4	4	4	20	4
6	5	5	6	6	4	26	5
7	6	6	5	5	5	27	6
8	5	5	5	6	6	27	6
9	6	7	5	5	5	28	7
10	7	8	7	7	7	36	8
11	8	9	8	9	8	42	9
12	9	11	9	8	9	46	10
13	9	10	10	9	10	48	11
14	10	12	11	10	11	54	12
15	11	13	12	11	12	59	13
16	12	14	13	12	13	64	14
17	13	15	14	13	14	69	15
18	14	16	15	14	15	74	16
19	15	17	16	15	16	79	17
20	16	18	17	16	17	84	18

As given in Tables 5 and 6, we considered class-wise (AD, PD, NC classes) statistics for CEN, IBA, GM and MCC performance metrics. This is helpful in finding the impact of class imbalance on the performance metrics. In Table 5 which is derived from Table 4, we report values of the RCI metric, and average of CEN, IBA, GM and MCC values. This is helpful in finding the best performing architectures based on these metrics.

A consolidated view of the best performing models is given in Table 8. As given in Table 7, we considered minimum of the balanced average of the individual class-based CEN values, while maximum of the balanced average of the individual class-based IBA, GM and MCC values. After that, as given in Table 8, we assigned a ranking to every method based on RCI, CEN, IBA, GM and MCC values. Then, we sum the ranking scores as given under overall score column in Table 8 and find the minimum among these scores. We form a new ranking system where minimum among these scores is given the best possible ranking.

As given in Tables 5 and 6, there is a variation between performances of different architectures which is natural. The architectures are correlating well with each other especially when it comes to learning the intricacies of AD class under CEN, IBA, GM and MCC metrics. It can be observed that average CEN metric is offering much greater variation in its values as compared to other performance metrics. On the other hand, RCI, average GM, average IBA and average MCC are offering better correlation among their values. For example, average CEN between AD/NC(SPECT)/PD classification with combined augmentations in the spatial domain using tenfold CV approach and AD/NC(SPECT)/PD classification with combined augmentations in the frequency domain using tenfold CV approach is ≈ 16% while other performance metrics offer a variation between 1 and 4%.

As given in Table 8, the best performing model has been found to be AD/NC(SPECT)/PD classification with random weak Gaussian blurred augmentation in the spatial domain using fivefold CV approach while the worst performing one has been found to be AD/NC(PET)/PD classification without augmentation in the frequency domain using tenfold CV approach. We can see that there is a strong correlation between the rankings provided by the individual performance metrics such as RCI, CEN, IBA, GM and MCC and the overall ranking for a method. We can see the advantages brought forth by the assessment based on multiple performance metrics rather than just one metric alone. Methods that employed augmentations clearly outperformed those that do not. In addition, we can see that methods that do not combine augmentations have a slight edge over those that combine them. We can also see that the spatial domain methods fared better in comparison to frequency domain counterparts which could be due to the fact that intensity values of image pixels in the spatial domain allow for a better representation of data than in frequency domain. Another point worth mentioning is that methods deploying less data have an edge over those that used more. Further, we can see that methods that employ NC(PET) class performed the worst and those that employed random weak Gaussian blurred augmentation fared better than their random zoomed in/out augmentation counterparts. We also noticed that methods that employed NC(PET) class are able to detect PD class instances perfectly while those that employed NC(SPECT) class are able to detect AD class instances perfectly. One reason for this accurate detection could be attributed to very weak correlation between the samples of both AD and PD subjects which are considered for this study.

In exploring the clinicopathological continuum between AD and PD subjects using PET and SPECT neuroimaging modalities and deep learning methods, we found that 3D-CNN architectures are an effective tool in discriminating the subjects of both these dementia types. The impairment of the brain in PD increases at a rapid pace due to a large number of factors such as age, tau pathology and lower CSF Aβ levels [18, 19]. Neuroimaging abnormalities in PD could be due to co-morbid AD developing memory impairment and dementia in patients [20].

In the experiments that we performed, we can completely discriminate between AD and PD subjects using different deep learning methods. However, there is a need for further research in this domain using more representative samples of AD and PD subjects as well as co-morbid AD/PD subjects using deep learning methods. In addition, there is a need for explaining the findings of a black box deep learning model in the exploration of clinicopathological continuum between AD and PD subjects.

## Conclusion

To conclude, we presented a study for the combined multiclass classification of AD, NC and PD subjects in the spatial and frequency domains using different data augmentation methods and a 3D-CNN architecture. The best performing model is AD/NC(SPECT)/PD classification with random weak Gaussian blurred augmentation in the spatial domain using fivefold CV approach while the worst performing model was AD/NC(PET)/PD classification without augmentation in the frequency domain using tenfold CV approach. We found that spatial domain methods have an edge over their frequency domain counterparts.

In the future, we are planning to extend this study using other frequency domain methods, data from other modalities, data augmentation techniques and novel architectures such as graph convolutional networks.

## Data Availability

The data used in the preparation of this manuscript is obtained from the Parkinson’s Progression Markers Initiative and Alzheimer’s Disease Neuroimaging Initiative databases.
